# Ca^2+^-stimulated ADCY1 and ADCY8 regulate distinct aspects of synaptic and cognitive flexibility

**DOI:** 10.3389/fncel.2023.1215255

**Published:** 2023-07-03

**Authors:** Ming Zhang, Hongbing Wang

**Affiliations:** Department of Physiology, Neuroscience Program, Michigan State University, East Lansing, MI, United States

**Keywords:** adenylyl cyclase, cAMP, calcium, depotentiation, hippocampus, LTD, LTP

## Abstract

The type 1 and 8 adenylyl cyclase (ADCY1 and ADCY8) exclusively account for Ca^2+^-stimulated cyclic AMP (cAMP) production and regulate activity-dependent synaptic modification. In this study, we examined distinct forms of synaptic plasticity in the hippocampus of *Adcy1*^−/−^ and *Adcy8*^−/−^ mice. We found that, at the Schaffer collateral-CA1 synapses, while the *Adcy8*^−/−^ mice displayed normal long-term potentiation (LTP) following various induction protocols with high-frequency stimulation (HFS), the *Adcy1*^−/−^ mice showed protocol-dependent deficits in LTP. We also found that long-term depression (LTD) requires ADCY1 but not ADCY8. Interestingly, both *Adcy1*^−/−^ and *Adcy8*^−/−^ mice showed defective synaptic depotentiation (i.e., activity-dependent reversal of LTP); the deficits in *Adcy8*^−/−^ mice were dependent on the induction protocol. Examination of spatial memory found that ADCY1 is required for the formation of both initial and reversal memory. ADCY8 is only required for reversal memory formation. These data demonstrate that ADCY1 and ADCY8 play distinct roles in regulating synaptic and cognitive flexibility that involves bidirectional modification of synaptic function.

## Introduction

While the central nervous system constantly receives information input from the changing environment, it undergoes dynamic synaptic modification to allow adaptive behavioral outcomes. Notably, the strength and efficacy of the synapses can be bidirectionally modified. On the one hand, certain neuronal activity (e.g., high-frequency firing) strengthens the synapses and induces long-term potentiation (LTP) (Nicoll, 2017). On the other hand, a different form of neuronal activity (e.g., low-frequency firing) weakens synaptic efficacy and induces long-term depression (LTD) (Collingridge et al., 2010). Moreover, the established synaptic potentiation can be reversed in an activity-dependent manner, leading to synaptic depotentiation (Wagner and Alger, 1996). It is evident that bidirectional synaptic modification naturally co-occurs with behavior adaptation such as learning and reversal learning (Whitlock et al., 2006; Clarke et al., 2010; Dong et al., 2012; Nabavi et al., 2014). The molecular mechanism that regulates synaptic and cognitive flexibility remains largely elusive.

The cAMP-mediated neuronal signaling pathway is highly conserved and regulates synaptic plasticity and learning and memory in invertebrates and vertebrates (Abel and Nguyen, 2008; Kandel, 2012). With regard to activity-dependent stimulation of cAMP signaling, the Ca^2+^-stimulated adenylyl cyclase (ADCY) is functionally positioned to integrate Ca^2+^ and cAMP signaling in excitable cells (Chen et al., 2022). Previous molecular and genetic studies have identified type 1 and type 8 ADCY (i.e., ADCY1 and ADCY8) as the only Ca^2+^-stimulated ADCY in the brain (Wong et al., 1999). Mice lacking both ADCY1 and ADCY8 (i.e., the *Adcy1*^−/−^*/Adcy8*^−/−^ double knockout mice) show impaired LTP, LTD, and depotentiation at the Shaffer-collateral CA1 synapses in the hippocampus (Wong et al., 1999; Zhang et al., 2011). The *Adcy1*^−/−^*/Adcy8*^−/−^ mice do not show learning-induced activation of CRE-mediated gene transcription (Sindreu et al., 2007; Zheng et al., 2012). Various forms of hippocampus-dependent memory, including spatial memory, passive avoidance memory, and contextual fear memory are impaired in the *Adcy1*^−/−^*/Adcy8*^−/−^ mice (Wong et al., 1999; Zhang et al., 2011).

Although *Adcy1*^−/−^ mice and *Adcy8*^−/−^ mice show normal *in vitro* CA1 LTP (Wong et al., 1999), it is not clear whether ADCY1 or ADCY8 alone is sufficient to support various aspects of synaptic and cognitive flexibility. ADCY1 and ADCY8 are not redundant Ca^2+^-stimulated ADCYs. Biochemical studies demonstrate that ADCY1 and ADCY8 are differentially regulated. While ADCY1 is regulated by both G protein-coupled receptors and Ca^2+^, ADCY8 is only regulated by Ca^2+^. Compared to ADCY1, ADCY8 is less sensitive to Ca^2+^. With an EC_50_ of ~100 nM by Ca^2+^, ADCY1 is partially and constitutively activated by basal Ca^2+^ in resting neurons and further activated in stimulated neurons. ADCY8 with an EC_50_ of ~500–800 nM by Ca^2+^ is likely inactive in resting neurons; its enzymatic activation mainly occurs in stimulated neurons (Chen et al., 2022). ADCY1 and ADCY8 show different contributions to Ca^2+^-stimulated cAMP production. In the hippocampus of *Adcy1*^−/−^ mice and *Adcy8*^−/−^ mice, the Ca^2+^-stimulated cAMP production is reduced by ~50 and ~30%, respectively (Wong et al., 1999).

In this study, we examined whether ADCY1 and ADCY8 have distinct functions in regulating synaptic flexibility. We examined *in vivo* LTP, LTD, and depotentiation at the Schaffer collateral-CA1 synapses in the hippocampus of *Adcy1*^−/−^ and *Adcy8*^−/−^ mice. We also examined cognitive flexibility with hippocampus-dependent spatial learning and reversal learning.

## Materials and methods

### Animals

This study used 2.5- to 3-month-old male C57BL/6 mice. Mice with ADCY1 and ADCY8 deficiency (i.e., *Adcy1*^−/−^ and *Adcy8*^−/−^) have been backcrossed with C57BL/6 mice for more than 20 generations. All mice were group housed (five or fewer per cage), had free access to food and water, and were relocated to clean housing cages once every week. The animal room was kept at 21.0 ± 1.0°C and with a 12 h light/dark cycle. All procedures have been reviewed and approved by the Institutional Animal Care and Use Committee (IACUC) of Michigan State University.

### Electrophysiology with anesthetized mice

*In vivo* electrophysiological recordings at the Schaffer collateral-CA1 synapses in the hippocampus were performed as described in our previous studies (Zhang et al., 2011; Zhang and Wang, 2013). Mice were first anesthetized by i.p. injection of Nembutal sodium (100 mg/kg) and then mounted to a stereotaxic frame (David Kopf Instruments). The body temperature was maintained at 37.0 ± 0.5°C by a heating system with feedback input from the mouse's anal temperature. A 95% oxygen was supplied to the mouse's snout during recording. The stimulating and recording electrodes (a pair of Teflon-coated wires, 50 um ID, 100 um OD; World Precision Instruments) were placed at the Schaffer collateral of the dorsal hippocampus (AP, 1.7–1.9 mm; ML, 1.7–1.9 mm; DV, and 1.6 −2.0 mm from the skull surface) and the ipsilateral stratum radiatum of CA1 (AP, 1.7–1.9 mm; ML, 1.2–1.3 mm; DV, and 1.5–1.9 mm from the skull surface), respectively. The electrophysiological signals were sampled at 20 kHz with a Powerlab 4/30 System (ADInstruments). Fine electrode location adjustment was made to obtain the strongest field excitatory postsynaptic potential (fEPSP). Once the acceptable waveforms of fEPSP were found, a stimulation intensity that evokes 50% of the maximal fEPSP was chosen to establish a stable fEPSP baseline. After a stable baseline was maintained for at least 30 min, various protocols were used to induce LTP, LTD, and depotentiation. Three different HFS (high-frequency stimulation) protocols were used to induce LTP: one train of 100 pulses at 100 Hz and two trains of 100 pulses at 100 Hz with either spaced (i.e., 5 min) or compressed (i.e., 1 min) inter-train interval (ITI). To induce LTD, low-frequency stimulation (LFS) consisting of 900 pulses at 1 Hz was used. To examine synaptic depotentiation, we first delivered 2xHFS (two trains of 100 pulses at 100 Hz with 1 min ITI) and then LFS (900 or 450 pulses at 1 Hz). The interval between the 2xHFS and LFS was 0.5, 5, 10, or 30 min. The fEPSP was collected at 0.03 Hz (i.e., once per 30 s); the slopes of four consecutive fEPSPs were averaged and presented in the figures to reveal synaptic responses once every 2 min.

### Behavioral examination

Hippocampus-dependent spatial learning and memory were examined with the Morris water maze as described in our previous studies (Zhang et al., 2011; Zhang and Wang, 2013). Mice were first trained to navigate in the water maze (1.2 m in diameter) and learn to escape from the water by landing on the hidden platform (10 cm in diameter, placed 1 cm below the surface of the water). During training, mice were trained for 2 trials per day with 4 h of inter-trial interval (ITI) for 6 days. On day 7, mice were subjected to the first probe test. During the probe test, the hidden platform was removed, and mice were allowed to search for 60 s. The time spent searching in each quadrant, which was arbitrarily assigned the “target quadrant” as the area of the hidden platform location, was recorded. The number of passes that a mouse made over the hidden platform location was also recorded. The mice were further trained with the hidden platform paradigm for an additional 6 days (from day 8 to 13), followed by the second probe test on day 14. The trained mice were then subjected to reversal learning, during which the hidden platform was moved to the opposite quadrant. The first phase of reversal learning was two trials per day for 4 days (from day 15 to 18). The third probe test was performed on day 19, followed by 4 days of additional reversal learning (from days 20 to 23). The fourth probe test was performed on day 24. For each trial during the hidden platform and reversal platform training, the mice were introduced to the water maze from random and various entry locations; the escape latency, which is the time spent to find and land on the platform, was recorded.

### Data collection and analysis

The experimenters were not blind to the genotype. Experiments with the wild-type and mutant mice were carried out in an interleaved fashion. All data are expressed as mean ± SEM. Two-way repeated measures ANOVA and one-way ANOVA followed by the *post hoc* Tukey test were used to determine statistical significance. Statistics and data plotting were performed using GraphPad Prism 7.0. The plotted data were labeled and arranged to make figures in Microsoft PowerPoint and converted to TIFF files.

## Results

### ADCY1 but not ADCY8 is required for LTP

We examined *in vivo* fEPSP responses at the Schaffer collateral-CA1 synapses in anesthetized mice. We found that, consistent with previous studies, the input–output (I/O) relation and paired-pulse facilitation (PPF) are normal in *Adcy1*^−/−^ mice (Zheng et al., 2016) (Supplementary Figure 1). *Adcy8*^−/−^ mice also showed normal I/O relation (Supplementary Figure 1A; genotype effect: *F*_2, 27_ = 0.06, *p* = 0.94; genotype x stimulation intensity interaction: *F*_14, 189_ = 0.34, *p* = 0.99) and PPF (Supplementary Figure 1B; genotype effect: *F*_2, 27_= 0.55, *p* = 0.59; genotype x inter-pulse interval interaction: *F*_18, 243_= 0.76, *p* = 0.74). Considering that *Adcy1*^−/−^ and *Adcy8*^−/−^ mice hippocampal neurons show normal mEPSC, AMPAR-mediated current, and NMDAR-mediated current (Gong et al., 2007), we expect that ADCY1 and ADCY8 deficiency does not affect basal neurotransmission.

**Figure 1:**
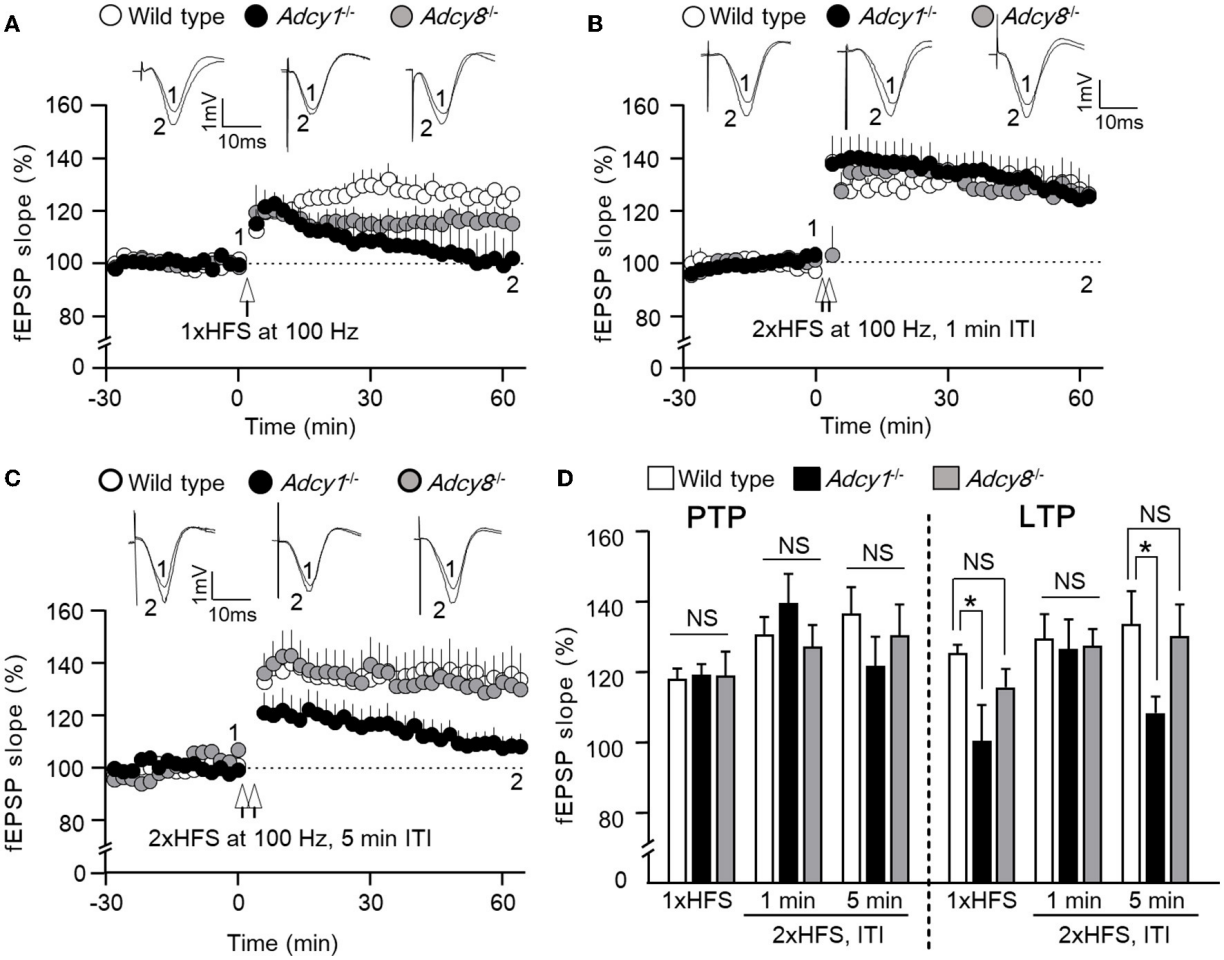
ADCY1 but not ADCY8 is required for HFS-induced LTP at the Schaffer collateral-CA1 synapses. Field excitatory post-synaptic potentials (fEPSP) were examined in anesthetized mice. Following the establishment of stable baseline fEPSP, different paradigms of high-frequency stimulation (HFS) were used to induce LTP. **(A)** LTP induced by a single HFS (1xHFS; 1 s duration at 100 Hz) in wild-type (*n* = 7), *Adcy1*^−/−^ (*n* = 5), and *Adcy8*^−/−^ (*n* = 8) mice. **(B)** LTP induced by 2 trains of HFS (2xHFS; 1 s duration at 100 Hz) with 1 min inter-train interval (ITI) in wild-type (*n* = 5), *Adcy1*^−/−^ (*n* = 5), and *Adcy8*^−/−^ (*n* = 6) mice. **(C)** LTP induced by 2xHFS (1 s duration at 100 Hz each) with 5 min ITI in wild-type (*n* = 5), *Adcy1*^−/−^ (*n* = 7), and *Adcy8*^−/−^ (*n* = 5) mice. **(D)** Average post-tetanic potentiation (PTP) during the first 10 min after the delivery of HFS in wild-type, *Adcy1*^−/−^, and *Adcy8*^−/−^ mice is shown in the left panel; average LTP during the last 10 min of recording is shown in the right panel. One-way ANOVA followed by *post hoc* Tukey multiple comparisons was used to determine statistical significance. *p < 0.05. NS, not significant.

We used three different high-frequency stimulation (HFS) protocols to induce LTP. While it was reported that a single HFS (100 Hz, 1 s) fails to induce LTP in the *Adcy1*^−/−^ mice (Zheng et al., 2016) (Figures 1A, D), we found normal LTP in the *Adcy8*^−/−^ mice (Figures 1A, D). Compared to the 125.5 ± 2.3% LTP in the wild-type mice, the *Adcy1*^−/−^ mice and *Adcy8*^−/−^ mice showed 100.4 ± 10.2% and 115.7 ± 5.2% potentiation, respectively (Figure 1D; genotype effect: *F*_2, 17_ = 4.08, *p* = 0.036).

When two trains of HFS [(2xHFS; 100 Hz for 1 s each) with 1 min inter-train interval (ITI)] were delivered, normal LTP was observed in both *Adcy1*^−/−^ mice (Zheng et al., 2016) and *Adcy8*^−/−^ mice (Figures 1B, D). Compared to the 129.6 ± 7.0% LTP in the wild-type mice, the *Adcy1*^−/−^ mice and *Adcy8*^−/−^ mice showed 126.5 ± 8.6% and 127.4 ± 4.8% potentiation, respectively (Figure 1D; genotype effect: *F*_2, 13_ = 0.05, *p* = 0.95).

When 2xHFS (100 Hz for 1 s each) with 5 min ITI was delivered, defective LTP was observed in *Adcy1*^−/−^ mice (Figures 1C, D); *Adcy8*^−/−^ mice showed normal LTP (Figures 1C, D). Compared to the 133.8 ± 9.3% LTP in the wild-type mice, the *Adcy1*^−/−^ mice and *Adcy8*^−/−^ mice showed 108.3 ± 4.7% and 130.2 ± 9.1% potentiation, respectively (Figure 1D; genotype effect: *F*_2, 14_ = 3.82, *p* = 0.047).

Following the delivery of HFS, the post-potentiation (PTP) was normal in *Adcy1*^−/−^ and *Adcy8*^−/−^ mice (Figure 1D). There was no significant difference in PTP after the 1xHFS (genotype effect: *F*_2, 17_ = 0.01, *p* = 0.99), the 2xHFS with 1 min interval (genotype effect: *F*_2, 13_ = 0.94, *p* = 0.42), and the 2xHFS with 5 min interval (genotype effect: *F*_2, 13_ = 0.86, *p* = 0.45). This indicates that the induction of LTP does not require ADCY1 and ADCY8.

In summary, these results show that ADCY8 is not required for LTP. Depending on the induction protocol, ADCY1 is required for certain forms of LTP.

### ADCY1 but not ADCY8 is required for LTD

**Figure 2:**
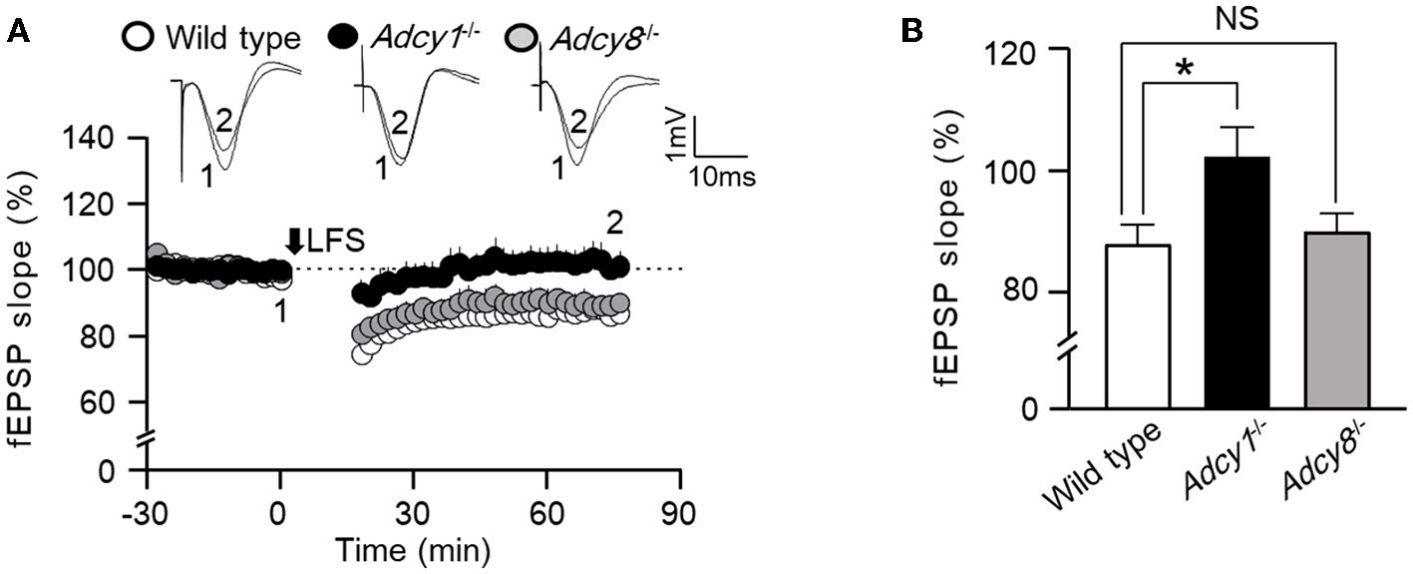
ADCY1 but not ADCY8 is required for the LFS-induced LTD at the Schaffer collateral-CA1 synapses. **(A)** Following the establishment of stable baseline fEPSP in the hippocampus of anesthetized mice, low-frequency stimulation (LFS; 900 pulses at 1 Hz) was used to induce LTD in wild-type (*n* = 6), *Adcy1*^−/−^ (*n* = 5), and *Adcy8*^−/−^ (*n* = 5) mice. **(B)** Average LTDs during the last 10 min of recording in the wild-type, *Adcy1*^−/−^, and *Adcy8*^−/−^ mice are compared. One-way ANOVA followed by *post hoc* Tukey multiple comparisons was used to determine statistical significance. *p < 0.05.

Synaptic efficacy can be bidirectionally modulated. In addition to synaptic potentiation, long-lasting synaptic weakening can also occur in an activity-dependent manner. We examined low-frequency stimulation (LFS)-induced *in vivo* LTD in anesthetized mice. We found that following 900 pulse stimulation at 1 Hz, both wild-type and *Adcy8*^−/−^ mice showed significant LTD (Figure 2). In contrast, *Adcy1*^−/−^ mice failed to develop measurable LTD (Figure 2). One-way ANOVA (Figure 2B; genotype effect: *F*_2, 13_ = 4.6, *p* = 0.032) followed by *post hoc* multiple comparisons revealed that, compared to the wild-type mice (83.81 ± 3.58%), LTD is defective in *Adcy1*^−/−^ mice (102.0 ± 4.8%, *p* < 0.05) but normal in *Adcy8*^−/−^ mice (89.5 ± 3.0%, *p* > 0.05).

### ADCY1 and ADCY8 are differentially required for synaptic depotentiation

**Figure 3:**
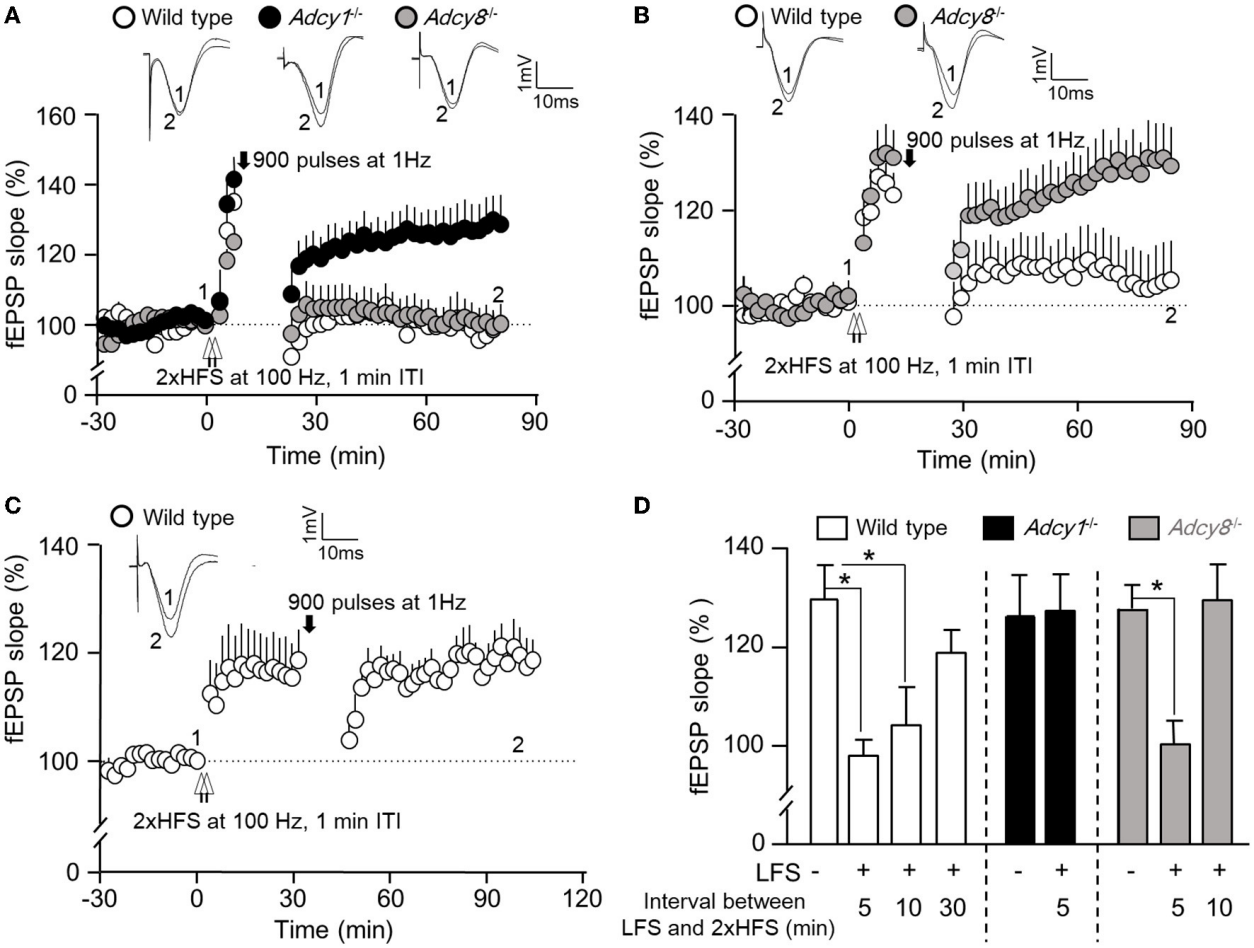
ADCY1 and ADCY8 are differentially required for activity-dependent reversal of synaptic potentiation. Following the establishment of stable baseline fEPSP in the hippocampus of anesthetized mice, two trains of HFS (2xHFS; 1 s duration at 100 Hz each, 1 min inter-train interval) were used to induce synaptic potentiation. Depotentiation was examined with relative changes in fEPSP after the delivery of LFS (900 pulses at 1 Hz). **(A)** LFS was delivered 5 min after the 2xHFS in wild-type (*n* = 5), *Adcy1*^−/−^ (*n* = 7), and *Adcy8*^−/−^ (*n* = 7) mice. **(B)** LFS was delivered 10 min after the 2xHFS in wild-type (*n* = 7) and *Adcy8*^−/−^ (*n* = 8) mice. **(C)** LFS was delivered 30 min after the 2xHFS in wild-type (*n* = 5) mice. **(D)** Summary of the LFS-induced depotentiation in the wild-type, *Adcy1*^−/−^, and *Adcy8*^−/−^ mice. The degrees of depotentiation by LFS delivered 5 min, 10 min, and 30 min after the 2xHFS are shown and compared. *p < 0.05, determined by one-way ANOVA followed by *post hoc* analysis with Tukey multiple comparisons.

An important aspect of synaptic flexibility is that the potentiated synaptic efficacy can be depotentiated (i.e., the reversal of synaptic potentiation). We used 2xHFS (2 × 100 Hz with 1 min ITI) to induce potentiation. When a 900-pulse LFS at 1 Hz was delivered 5 min after the 2xHFS, the wild-type (97.9 ± 4.3% of the baseline fEPSP) and *Adcy8*^−/−^ mice (100.2 ± 4.8% of the baseline fEPSP) showed significant depotentiation (Figures 3A, D). In contrast, LFS failed to reverse the synaptic potentiation in the *Adcy1*^−/−^ mice, which still showed LTP (127.2 ± 7.4% of the baseline fEPSP) (Figures 3A, D). These data demonstrate that ADCY1 but not ADCY8 is required for activity-dependent synaptic depotentiation.

It is known that depotentiation is achieved only when the LFS is delivered shortly after the HFS. When potentiation is consolidated, it is resistant to depotentiation (Huang and Hsu, 2001). We examined the effect of delayed delivery of LFS. We found that a 900-pulse LFS delivered 10 min after the 2xHFS was sufficient to cause depotentiation in the wild-type mice (104.2 ± 7.9% of the baseline fEPSP) but not *Adcy8*^−/−^ mice (129.4 ± 7.3% of the baseline fEPSP) (Figures 3B, D). A further delayed LFS delivered 30 min after the 2xHFS failed to cause depotentiation in the wild-type mice (118.8 ± 4.6% of the baseline fEPSP) (Figures 3C, D). Collectively, as summarized in Figure 3D, delayed delivery of LFS is less effective in reversing the previously established potentiation. ADCY8 is required for the depotentiation of the partially consolidated potentiation. ADCY1 is required for the depotentiation of even the freshly potentiated synapses.

**Figure 4:**
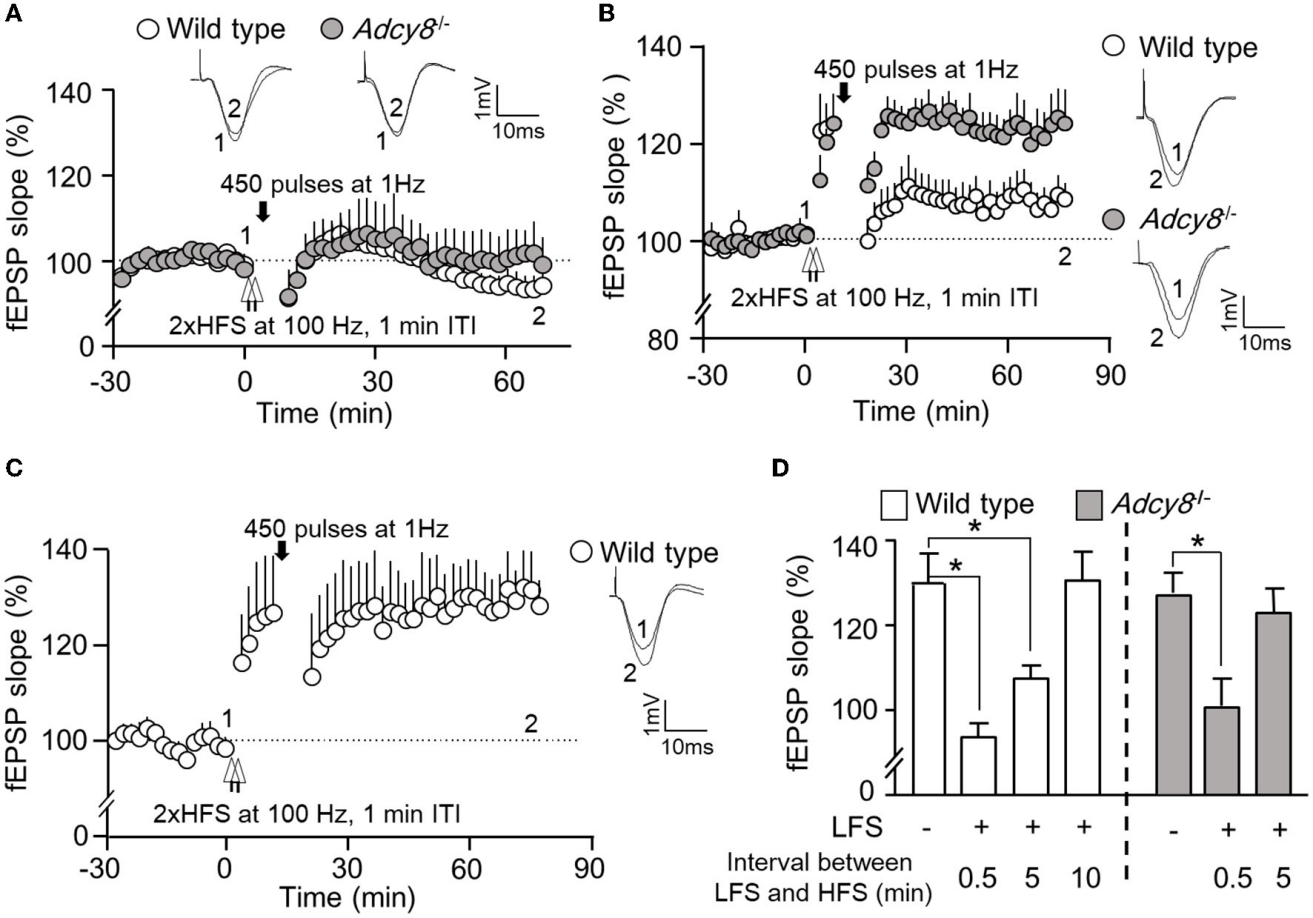
Activity-dependent reversal of synaptic potentiation by LFS consisting of 450 pulses of stimulation at 1 Hz. Following the establishment of stable baseline fEPSP in the hippocampus of anesthetized mice, two trains of HFS (2xHFS; 1 s duration at 100 Hz, 1 min inter-train interval) were used to induce synaptic potentiation. Depotentiation was examined with relative changes in fEPSP after the delivery of LFS (450 pulses at 1 Hz). **(A)** LFS was delivered 0.5 min after the 2xHFS in wild-type (*n* = 5) and *Adcy8*^−/−^ (*n* = 5) mice. **(B)** LFS was delivered 5 min after the 2xHFS in wild-type (*n* = 8) and *Adcy8*^−/−^ (*n* = 6) mice. **(C)** LFS was delivered 10 min after the 2xHFS in wild-type (*n* = 5) mice. **(D)** Summary of the LFS-induced depotentiation in the wild-type and the *Adcy8*^−/−^ mice. The degrees of depotentiation by LFS delivered 0.5 min, 5 min, and 10 min after the 2xHFS are shown and compared. *p < 0.05, determined by one-way ANOVA followed by *post hoc* analysis with Tukey multiple comparisons.

We further examined whether depotentiation is dependent on the intensity of LFS. We used 450 stimulations at 1 Hz as a relatively weaker LFS to induce depotentiation. When a 450-pulse LFS was delivered 0.5 min after the 2xHFS, it caused significant depotentiation in both wild-type and *Adcy8*^−/−^ mice (Figures 4A, D; 93.7 ± 3.3% in the wild-type mice and 100.7 ± 6.4% in the *Adcy8*^−/−^ mice). The 450-pulse LFS delivered 5 min after the 2xHFS caused depotentiation in the wild-type mice (107.6 ± 3.2% of the baseline fEPSP) but not *Adcy8*^−/−^ mice (123.2 ± 5.7% of the fEPSP) (Figures 4B, D). We noticed that, while a 900-pulse LFS delivered 10 min after the 2xHFS caused depotentiation in the wild-type mice (Figures 3B, D), a 450-pulse LFS delivered 10 min after the 2xHFS failed to reverse the potentiation in the wild-type mice (118.8 ± 4.6% of the baseline fEPSP) (Figures 4C, D). Our results, as summarized in Figures 3D, 4D, demonstrate that compared to the reversal of the freshly established potentiation, the reversal of the aged and partially consolidated potentiation requires higher activity input. The activity threshold (i.e., the degree of LFS) to cause depotentiation is higher in neurons with ADCY8 deficiency.

### ADCY1 but not ADCY8 is required for spatial memory formation

**Figure 5:**
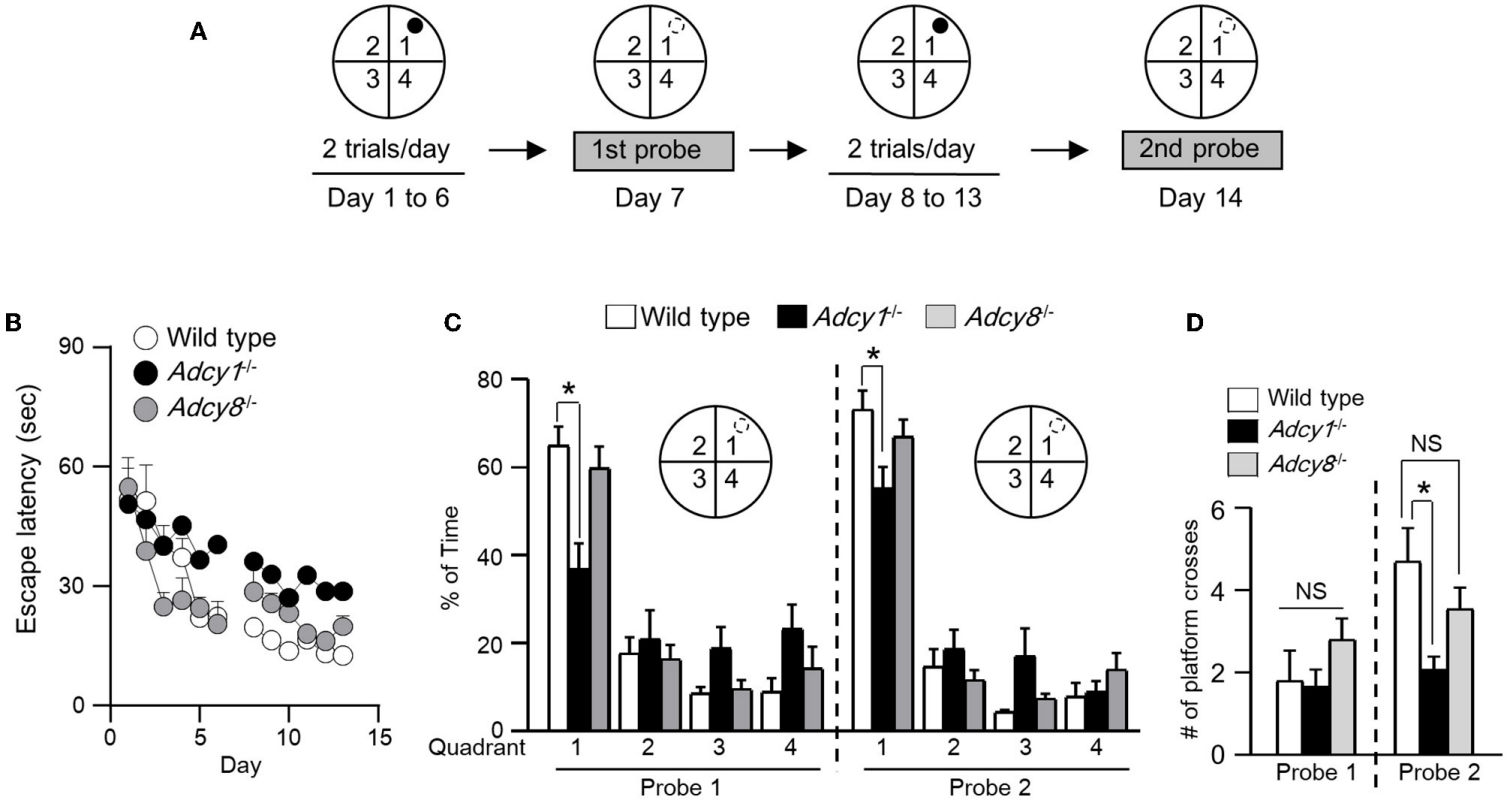
ADCY1 regulates spatial learning and memory formation. **(A)** Behavior procedure. Wild-type (*n* = 10), *Adcy1*^−/−^ (*n* = 12), and *Adcy8*^−/−^ (*n* = 11) mice were subjected to hidden platform training in the Morris water maze. The water maze was arbitrarily divided into four quadrants; the hidden platform was placed in quadrant 1 as indicated by a filled circle. Mice were trained with two trials/day for 6 days and subjected to the first probe test on day 7. The mice were then further trained twice per day for 6 additional days and subjected to the second probe test on day 14. During the probe tests, the hidden platform was removed; the hidden platform location is indicated by an open circle outlined with broken lines. **(B)** During training, the time mice spent to escape from the water and land on the hidden platform was recorded and expressed as escape latency. **(C, D)** During the probe tests, the percentage of total time spent in searching each quadrant **(C)** and the number of crosses over the hidden platform location **(D)** were recorded. *p < 0.05, determined by Tukey multiple comparisons following two-way repeated measures ANOVA **(C)** or one-way ANOVA **(D)**. NS, not significant.

To determine whether synaptic flexibility may reflect certain aspects of cognitive flexibility, we examined hippocampus-dependent spatial memory. With the Morris water maze task, we first trained the animals to learn the spatial cues to escape from the water and land on the hidden platform (Figure 5A). Across the training sessions, the *Adcy1*^−/−^ but not *Adcy8*^−/−^ mice showed defective learning and took more time to find the hidden platform (Figure 5B; genotype effect: *F*_2, 30_ = 4.4, *p*=0.021; training/time effect: *F*_11, 330_ = 14.3, *p* < 0.00001; genotype x training interaction: *F*_22, 330_= 1.4, *p* = 0.14). After 6 days of hidden platform training, the wild-type and *Adcy8*^−/−^ mice but not the *Adcy1*^−/−^ mice spent more time searching the target quadrant during the first probe test (Figure 5C; genotype effect: *F*_2, 30_= 0.9, *p* = 0.44; quadrant effect: *F*_3, 90_= 40.0, *p* < 0.0001; genotype × quadrant interaction: *F*_6, 90_= 3.8, *p* = 0.002). After additional 6 days of hidden platform training, all three groups of mice showed a preference for searching the target quadrant during the second probe test (Figure 5C; genotype effect: *F*_2, 30_= 1.7, *p* = 0.19; quadrant effect: *F*_3, 90_= 109.2, *p* < 0.0001; genotype x quadrant interaction: *F*_6, 90_= 2.6, *p* = 0.023). However, the wild-type mice spent more time in the target quadrant than the *Adcy1*^−/−^ but not the *Adcy8*^−/−^ mice (Figure 5C). The number of platform crosses which reflects the degree of precise spatial navigation, was comparable among the different groups during the first probe test (Figure 5D; *F*_2, 30_= 1.3, *p* = 0.28). In the second probe test, the wild-type mice showed increased platform crossing than the *Adcy1*^−/−^ but not the *Adcy8*^−/−^ mice (Figure 5D; *F*_2, 30_= 5.5, *p* = 0.009). The defective spatial learning and memory formation in the *Adcy1*^−/−^ mice is not due to an alteration of motor function. These mice with different genotypes showed similar swimming speeds (Supplementary Figure 2; *F*_2, 30_= 1.132, *p* = 0.336).

### ADCY1 and ADCY8 are differentially required for spatial memory reversal

**Figure 6:**
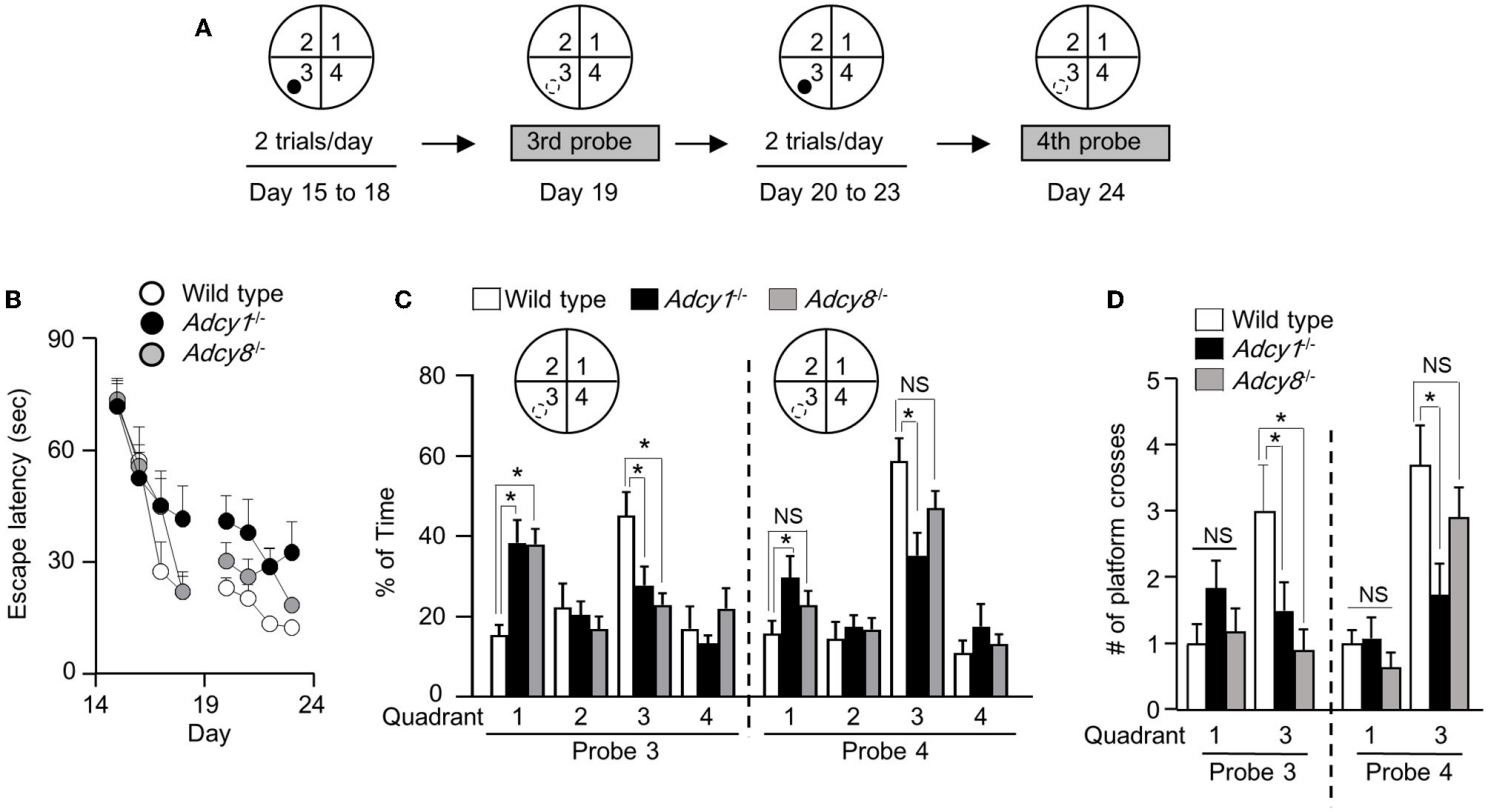
Effective old memory suppression and new spatial memory establishment require ADCY1 and ADCY8. **(A)** Behavior procedure. After completion of the hidden platform training/testing, the same mice were subjected to reversal platform training, during which the platform (indicated by a filled circle) was moved to the opposite quadrant (i.e., quadrant 3). The mice were trained with two trials/day for 4 days (from day 15 to 18) and subjected to the third probe test on day 19. The mice were then further trained twice per day for 4 additional days (from day 20 to 23) and subjected to the fourth probe test on day 24. During the probe tests, the hidden platform was removed; the hidden platform location is indicated by an open circle outlined with broken lines. **(B)** During the reversal training, the escape latency to land on the platform at the new reversal location was recorded. **(C, D)** During the probe tests, the percentage of total time spent in searching each quadrant **(C)** and the number of crosses over the old and new hidden platform location **(D)** were recorded. *p < 0.05, determined by Tukey multiple comparisons following two-way repeated measures ANOVA. NS, not significant.

We next subjected the trained animals to the reversal platform training, during which the hidden platform was moved to a new quadrant (i.e., quadrant 3 as indicated in Figure 6A) in contrast to the initial target quadrant (i.e., quadrant 1, as indicated in Figure 5A). During the reversal learning process, the animals are expected to learn the new platform location and, in the meantime, learn that the old platform location is irrelevant and obsolete. Consequently, new memory is established along with the suppression of the old memory.

Across all reversal platform training sessions, all three groups showed comparable improvement in escape latency (Figure 6B; genotype effect: *F*_2, 30_= 2.1, *p* = 0.15; training/time effect: *F*_7, 210_= 39.9, *p* < 0.00001; genotype x training interaction: *F*_14, 210_= 1.6, *p* = 0.096). After 4 days of reversal learning, only the wild-type but not the *Adcy1*^−/−^ and *Adcy8*^−/−^ mice spent more time searching the new target quadrant during the third probe test (Figure 6C; genotype effect: *F*_2, 30_= 0.85, *p* = 0.44; quadrant effect: *F*_3, 90_= 6.3, *p* = 0.0006; genotype x quadrant interaction: *F*_6, 90_= 4.2, *p* = 0.0009). The *Adcy1*^−/−^ and *Adcy8*^−/−^ mice still spent more time searching the old target quadrant (Figure 6C). After 4 days of additional reversal learning, both the wild-type and *Adcy8*^−/−^ mice showed a preference for the new but not the old platform quadrant during the fourth probe test; the *Adcy1*^−/−^ mice still failed to show preference in searching the new target quadrant (Figure 6C; genotype effect: *F*_2, 30_= 1.5, *p* = 0.25; quadrant effect: *F*_3, 90_= 27.4, *p* < 0.0001; genotype x quadrant interaction: *F*_6, 90_= 2.7, *p* = 0.02). In the third probe test, only the wild-type mice showed more crosses over the new platform location (Figure 6D; genotype effect: *F*_2, 30_= 3.4, *p* = 0.046; platform effect: *F*_1, 30_= 1.3, *p* = 0.26; genotype × platform interaction: *F*_2, 30_= 3.4, *p* = 0.046). In the fourth probe test, the wild-type and *Adcy8*^−/−^ but not the *Adcy1*^−/−^ mice showed more crossings over the new platform location (Figure 6D; genotype effect: *F*_2, 30_= 2.7, *p* = 0.087; platform effect: *F*_1, 30_= 33.1, *p* < 0.0001; genotype × platform interaction: *F*_2, 30_= 3.7, *p* = 0.036).

## Discussion

Although the functional importance of synaptic and cognitive flexibility is recognized, the underlying mechanism remains largely unknown. Regarding the role of cAMP signaling, it is known that the reduction of PKA (cAMP-dependent protein kinase A) impairs LTP and spatial memory (Abel et al., 1997). Intriguingly, PKA activity is not only required for activity-dependent synaptic potentiation but also supports LTD and depotentiation (Brandon et al., 1995), but also see Malleret et al. (2010). The function of PKA in regulating the reversal of spatial memory has not been investigated. We determined how ADCY1 and ADCY8, which may directly activate PKA in an activity-dependent manner, regulate synaptic and cognitive flexibility.

Previous studies found that the *in vitro* CA1 LTP in acute hippocampal slices is normal in *Adcy1*^−/−^ and *Adcy8*^−/−^ mice (Wu et al., 1995; Wong et al., 1999). In this study, we found that, while the *Adcy8*^−/−^ mice show normal *in vivo* LTP, ADCY1 is required for certain forms of LTP. Complementing our previous findings (Zheng et al., 2016), we found that ADCY1 is required for LTP induced by 2xHFS with longer (i.e., 5 min) but not shorter intervals (i.e., 1 min). This is consistent with the speculation that the establishment of LTP engages a rapid activation of calmodulin-dependent protein kinase II (CaMKII) and a slightly delayed activation of PKA (Woo et al., 2003; Kim et al., 2010). Interestingly, it is more difficult to establish LTP in the *Adcy1*^−/−^ mice, but once the LTP is established, the potentiation is more stable and resistant to the activity-dependent reversal. At the behavior level, it is more difficult to establish spatial memory in the *Adcy1*^−/−^ mice, but once the memory is established, it is more stable and resistant to reversal learning. In contrast, deficiency in ADCY8, whose enzymatic activity is less responsive to calcium than ADCY1 (Wong et al., 1999; Chen et al., 2022) has no effect on LTP and spatial memory formation.

One complication is that HFS at 100 Hz also causes LTD in CA1 inhibitory neurons (iLTD), which may, in turn, affect synaptic potentiation in CA1 excitatory neurons (Patenaude et al., 2003; Castillo et al., 2011). Single neuron RNAseq studies revealed that *Adcy1* mRNA is expressed in excitatory but not inhibitory neurons in the hippocampus; *Adcy8* mRNA expression is higher in excitatory neurons than in inhibitory neurons in the hippocampus (Yue et al., 2014) (https://brainrnaseq.org). We speculate that the defective LTP in *Adcy1*^−/−^ mice reflects altered plasticity in excitatory neurons. Although *Adcy8*^−/−^ mice show normal LTP, future studies with cell-type specific *Adcy8* deficiency will more precisely examine the potential impact of cAMP-regulated iLTD through ADCY8 (Chevaleyre et al., 2007).

Our data along with previous research suggest distinct functions of the Ca^2+^-stimulated ADCY in regulating plasticity within the hippocampal trisynaptic circuit. While ADCY1 but not ADCY8 is required for LTP at the Schaffer collateral/CA1 synapse, both ADCY1 and ADCY8 are required for LTP at the mossy fiber/CA3 synapse (Villacres et al., 1998; Wang et al., 2003). The perforant path/dentate gyrus (DG) LTP is normal in *Adcy1*^−/−^ and *Adcy8*^−/−^ mice (Villacres et al., 1998; Wang et al., 2003). With regard to postsynaptic vs. presynaptic regulation of LTP, we found that ADCY1 and ADCY8 are expressed in both dendrites and axons of hippocampal neurons (Wang et al., 2002, 2003). Notably, ADCY1 and ADCY8 are enriched at the postsynaptic density and the presynaptic active zone, respectively (Conti et al., 2007). The region-specific presynaptic function of ADCY8 but not ADCY1 is recognized (Villacres et al., 1998; Wang et al., 2003). The PPF (paired-pulse facilitation) in *Adcy8*^−/−^ mice is altered in CA3 but not CA1 neurons (Wong et al., 1999; Wang et al., 2003).

Cellular mechanisms underlying synaptic depotentiation have not been extensively investigated. It is conceivable that freshly established potentiation is less stable and more susceptible to re-modification. Our data show that there is a time window as well as an activity threshold (i.e., 900-vs. 450-pulse LFS) to allow effective depotentiation *in vivo*. Strikingly, natural behavior, such as novelty exploration, can reverse freshly established but not consolidated synaptic potentiation in freely moving animals (Xu et al., 1998). Activity level over certain threshold values is also necessary for depotentiation, which is practically achieved with 30 min but not 10 min novelty exploration (Qi et al., 2013). Our results suggest that the Ca^2+^-stimulated cAMP signaling may shape and control the time dependency and threshold. ADCY1 and ADCY8 deficiency makes the potentiated synapses more stable and more resistant to depotentiation. Their downstream targets that directly regulate synaptic efficacy remain to be identified.

Little is known about whether and how synaptic flexibility correlates with behavior flexibility. Previous studies have observed a correlation between LTD and reversal memory establishment (Nicholls et al., 2008; Malleret et al., 2010; Zhang and Wang, 2013). Our data identified an interesting correlation between the reversal of the previously established synaptic potentiation (i.e., depotentiation) and the reversal of the previously established spatial memory. The *Adcy1*^−/−^ mice lack depotentiation and memory reversal. In the *Adcy8*^−/−^ mice, the degree of depotentiation depends on the intensity of LFS. While 450 pulses at 1 Hz failed to depotentiate, 900 pulses reversed the previously established potentiation in the *Adcy8*^−/−^ mice. In parallel, the *Adcy8*^−/−^ mice still searched the old platform location after 4 days of reversal platform training. Their old memory was significantly depreciated after 8 days of reversal training. Considering the function of other brain regions (e.g., prefrontal cortex) in regulating cognitive flexibility, future studies with specific CA1 alterations are required to examine the causal function of depotentiation in regulating reversal learning.

Genome-wide association study (GWAS) and linkage study identified *Adcy1* (Sundararajan et al., 2018) and *Adcy8* (Avramopoulos et al., 2004; Zhang et al., 2010) as genetic risk factors for schizophrenia and bipolar disorder, respectively. The level of *Adcy8* mRNA is altered in postmortem brain samples collected from schizophrenia and bipolar disorder patients (Guan et al., 2019). In addition to affective symptoms and dysregulated mood, cognitive impairment is also prevalent and, to a significant degree, impacts mental health and daily functioning in patients with psychiatric disorders including schizophrenia and bipolar disorder (Marder and Fenton, 2004; Martinez-Aran et al., 2004; Keefe et al., 2014). Notably, dysfunctional cognitive flexibility, which is mainly examined by reversal learning tasks in clinical settings, is an outstanding aspect of cognitive disability in schizophrenia and bipolar disorder (McKirdy et al., 2009; Wegbreit et al., 2016). Interestingly, selective depotentiation defects have been reported in both the neurodevelopmental (Sanderson et al., 2012) and genetic mouse models (Shamir et al., 2012) of schizophrenia. Our present study revealed that ADCY8 deficiency selectively affects depotentiation but not LTP and LTD. ADCY8 deficiency also selectively affects the establishment of the reversal but not the initial spatial memory.

In summary, this study demonstrates that ADCY1 and ADCY8 are not functionally redundant. They regulate distinct aspects of synaptic and cognitive flexibility. Our data also reveal that the depression of naïve synapses (i.e., LTD) and the depression of the potentiated synapses (i.e., depotentiation) are differentially regulated.

## Data availability statement

The original contributions presented in the study are included in the article/Supplementary material, further inquiries can be directed to the corresponding author.

## Ethics statement

The animal study was reviewed and approved by Institutional Animal Care and Use Committee, Michigan State University.

## Author contributions

HW initiated the study. MZ performed the study and analyzed the data. MZ and HW wrote the manuscript. Both authors contributed to the article and approved the submitted version.

## Supplementary material

Supplementary Figure 1 ADCY1 and ADCY8 deficiency do not affect basal neural transmission and short-term plasticity. Wild-type (*n* = 15), *Adcy1*^−/−^ (*n* = 13), and *Adcy8*^−/−^ (*n* = 13) mice were examined. **(A)** Basal neurotransmission was examined by the fEPSP responses to different stimulation intensities. **(B)** Short-term plasticity was examined by paired-pulse facilitation (PPF) triggered by paired stimulations with different inter-pulse intervals. The insets are representative of fEPSP stimulated by two pulses with 80 ms interval.

Supplementary Figure 2 ADCY1 and ADCY8 deficiency does not affect locomotion in the water maze training. The swimming speed, as determined by the average value during the hidden platform trials, is comparable among the wild-type (*n* = 10), *Adcy1*^−/−^ (*n* = 12), and *Adcy8*^−/−^ (*n* = 11) mice. NS: not significant, determined by one-way ANOVA.
