# Supplementary figures and images for: Ca2+-stimulated ADCY1 and ADCY8 regulate distinct aspects of synaptic and cognitive flexibility
Source: Front Cell Neurosci. 2023 Jul 3;17:1215255. doi: 10.3389/fncel.2023.1215255 (PMC10351016; doi:10.3389/fncel.2023.1215255)

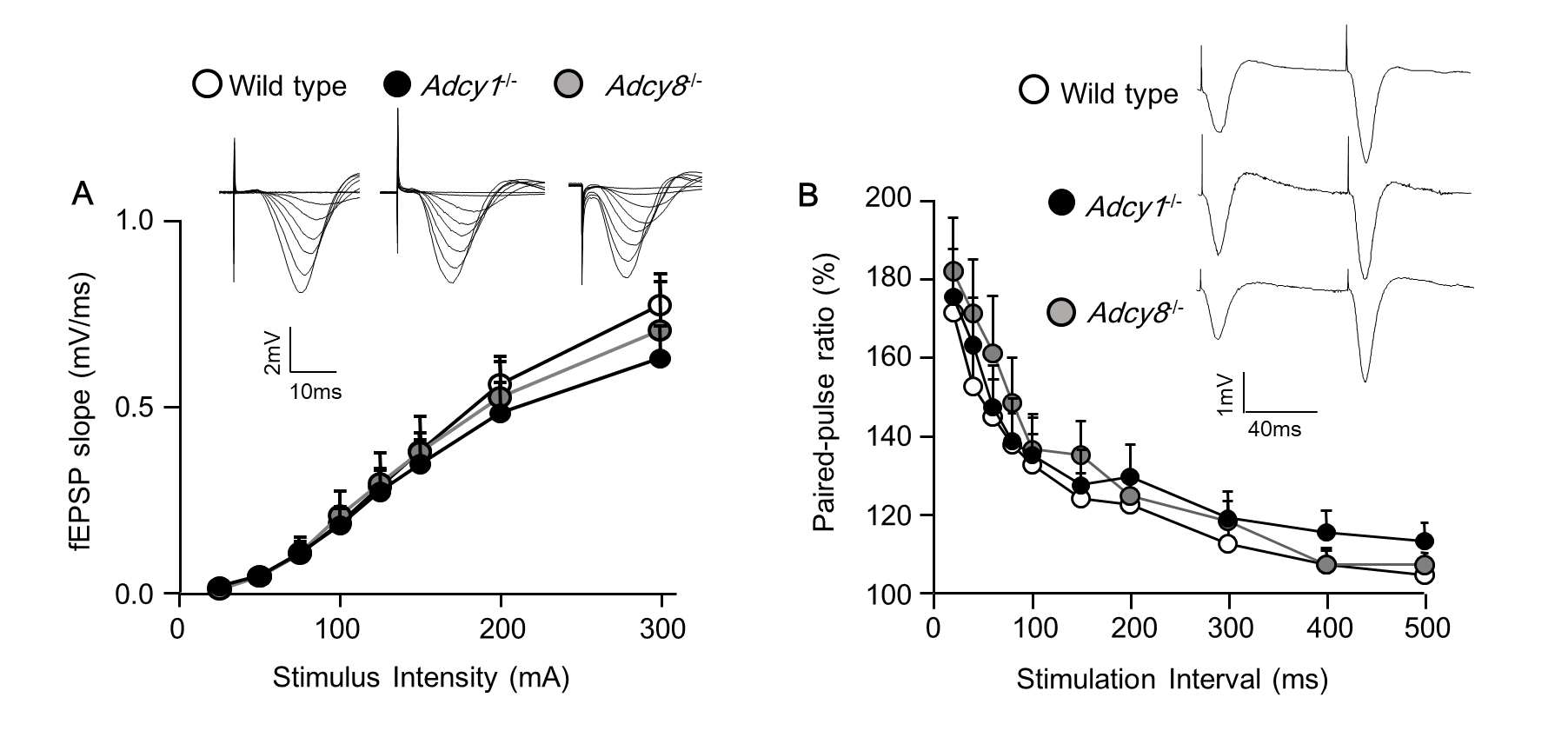

Supplement: Supplementary Figure 1 — ADCY1 and ADCY8 deficiency do not affect basal neural transmission and short-term plasticity. Wild-type (n = 15), Adcy1−/− (n = 13), and Adcy8−/− (n = 13) mice were examined. (A) Basal neurotransmission was examined by the fEPSP responses to different stimulation intensities. (B) Short-term plasticity was examined by paired-pulse facilitation (PPF) triggered by paired stimulations with different inter-pulse intervals. The insets are representative of fEPSP stimulated by two pulses with 80 ms interval. [file Image_1.TIF]

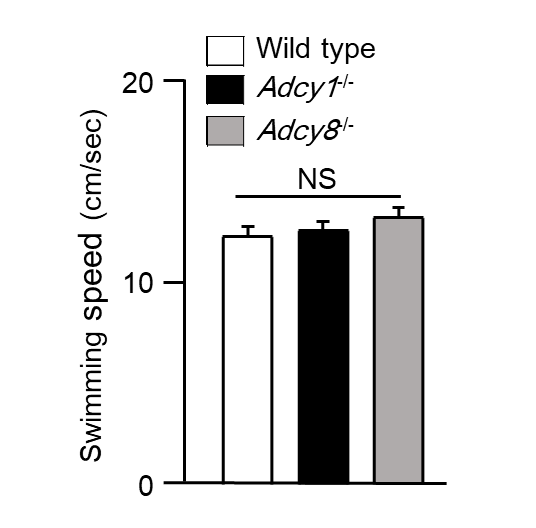

Supplement: Supplementary Figure 2 — ADCY1 and ADCY8 deficiency does not affect locomotion in the water maze training. The swimming speed, as determined by the average value during the hidden platform trials, is comparable among the wild-type (n = 10), Adcy1−/− (n = 12), and Adcy8−/− (n = 11) mice. NS: not significant, determined by one-way ANOVA. [file Image_2.TIF]
